# Supplementary material for: Mis-regulation of Zn and Mn homeostasis is a key phenotype of Cu stress in Streptococcus pyogenes
Source: Metallomics. 2023 Oct 17;15(11):mfad064. doi: 10.1093/mtomcs/mfad064 (PMC10644519; doi:10.1093/mtomcs/mfad064)
Supplement: mfad064_Supplemental_File [file mfad064_supplemental_file.docx]

**Mis-regulation of Zn and Mn homeostasis is a key phenotype of Cu stress in *Streptococcus pyogenes*.**

YoungJin Hong^1^*, Eilidh S Mackenzie^2^*, Samantha J Firth^1^*, Jack RF Bolton^1^*, Louisa J Stewart^1^*, Kevin J Waldron^2^, and Karrera Y Djoko^1#^

**SUPPLEMENTARY MATERIALS**

**Table S1.** List of GAS strains used in this study.

| Bacterial strains | Description | Reference |
| --- | --- | --- |
| 5448 (WT) | Invasive M1T1 strain | [1] |
| 5448Δ*copA* | Δ*copA*::*kan* deletion mutant | [2] |
| 5448Δ*adcAI/II* | Δ*adcA*::*kan* Δ*adcAII*::*aad* double deletion mutant | [3] |
| 5448Δ*adcBC* | Δ*adcBC*::*kan* deletion mutant | [3] |
| 5448Δ*mtsABC* | Δ*mtsABC::aphA-3* deletion mutant | [4] |
| 5448Δ*mtsABC::mtsABC+* | *ΔmtsABC* marker rescue mutant | [4] |

**Table S2.** List of primers used in this study. Primers were designed using the genome sequence of *S. pyogenes* MGAS5005 as template (NCBI GenBank Accession No CP000017.2).

| **Amplicon name** | **Primer name** | **Sequence** | **Locus Tag of target gene** | **Amplicon size (bp)** |
| --- | --- | --- | --- | --- |
| adcAI | adcAI-qPCR-F | GGAACTGGTAACATGCTCTTGG | M5005_Spy0543 | 109 |
|  | adcAI-qPCR-R | CATGGTTGTGTCCTTCTTCGC |  |  |
| adcAII | adcAII-qPCR-F | AGGTTGATGTGTTTGAAGCG | M5005_Spy1711 | 106 |
|  | adcAII-qPCR-R | GGGTCATAAAGTGTCGCAGG |  |  |
| adcC | adcC-qPCR-F | CCATCCACCGTTTACGAGTTTG | M5005_Spy0078 | 99 |
|  | adcC-qPCR-R | GCTTGCTTGCACATGCTCTTC |  |  |
| copA | copA-qPCR-F | TGGTCTCAGGTCTTGTGGTC | M5005_Spy1405 | 97 |
|  | copA-qPCR-R | GTTGCAGAAATGGAGGAAGCC |  |  |
| copZ | copZ-qPCR-F | GCAATCGGTCCAGGTAAATTTGG | M5005_Spy1404 | 92 |
|  | copZ-qPCR-R | TGGTATCCTTCAAAGCACGC |  |  |
| holB | holB-qPCR-F | CATAGTAATCGTAGTGGGTTTCGC | M5005_Spy1835 | 91 |
|  | holB-qPCR-R | CATTTGAAGTCTGCTTAGAACGTG |  |  |
| mtsC | mtsC-qPCR-F | ACTCTGTCATTAAAGGAGATACGGC | M5005_Spy0370 | 100 |
|  | mtsC-qPCR-R | AAGTCCGTCGAACTATTGGC |  |  |
| siaA | siaA-qPCR-F | TGTTGAGGGCATGTACCAGTC | M5005_Spy1528 | 93 |
|  | siaA-qPCR-R | TAGTCCTGGTATTGCTGGCG |  |  |
| fhuA | fhuA-qPCR-F | TTCTTACGGACGTTTTCCCC | M5005_Spy0324 | 104 |
|  | fhuA-qPCR-R | CATAGGCCATGACATTGGTGG |  |  |
| gapA | gapA-check-F | GTAGTTAAAGTTGGTATTAACGG | M5005_Spy0233 | 1008 |
|  | gapA-check-R | TTTAGCAATTTTTGCGAAGTACTCA |  |  |

**Figure S1. Effects of Cu treatment on expression levels of (A) *adcAII*, (B) *adcC*, and (C) *fhuA*.** The GAS 5448∆*copA* mutant strain was cultured with or without added Cu (5 µM) for *t =* 4, 6, or 8 h (*N* = 4). mRNA levels of target genes in Cu-supplemented cultures (+Cu) were determined by qRT-PCR and normalised to those in the corresponding unsupplemented samples (‑Cu) that were cultured for the same time periods. Dotted horizontal lines represent the sensitivity limit of the assay (log_2_FC = ± 0.5). Data from individual replicates are shown. Lines indicate means. Expression levels of *adcAII* (*P* = 0.012) were time-dependent but not those of *adcC* (*P* = 0.094) or *fhuA* (*P* = 0.98).

**Figure S2. Effects of Cu treatment on cellular levels of (A) Zn, (B) Mn, (C) Fe, and (D) Cu, sampled at *t* = 4 h.** The GAS 5448∆*copA* mutant strain was cultured with supplemental Cu (0, 1, or 5 µM) for *t* = 4 h (*N* = 5). Total cellular levels of all metals were measured by ICP MS and normalised to total cellular protein content. Data from individual replicates are shown. Columns indicate means. Error bars indicate SD. Cu treatment influenced cellular levels of Cu (*P* < 0.0001) but not those of Zn, Mn, or Fe (*P* = 0.12, 0.11, or 0.86 respectively).

**Figure S3. Effects of co-supplemental Zn and Mn on cellular levels of (A) Zn and (B) Mn.** The GAS 5448∆*copA* mutant strain was cultured with added Cu (0, 1, or 5 µM) with or without Zn or Mn (0, 0.5, or 5 µM) for *t* = 8 h (*N* = 5). Total cellular levels of Zn and Mn were measured by ICP MS and normalised to total cellular protein content. Note that the H_2_O data are also shown in Figure 3. Data from individual replicates are shown. Columns indicate means. Error bars represent SD. Co-supplemental Zn led to an increase in cellular Zn levels (*P* = 0.0007 and <0.0001, respectively, for 0.5 and 5 µM Zn) but not Mn levels (*P* = 1.0 and 0.86, respectively, for 0.5 and 5 µM Zn). Co-supplemental Mn led to an increase in cellular Mn levels (*P* <0.0001 each for 0.5 and 5 µM Mn) but not Zn levels (*P* = 0.93 and 0.75, respectively, for 0.5 and 5 µM Mn).

**Figure S4. Effects of co-supplemental Zn or Mn on Cu-dependent expression of (A) *adcAI* or (B) *mtsC*.** The GAS 5448∆*copA* mutant strain was cultured with added Cu (0 – 5000 nM), with or without added Zn or Mn (5 µM each) for *t* = 8 h (*N* = 3). Levels of *adcAI* or *mtsC* mRNA in these samples were determined by qRT-PCR and normalised to expression of *holB* as the control. Data from individual replicates are shown. Lines indicate means. Growth in the presence of Cu alone (*P* < 0.0001) or Zn alone (*P* < 0.0001) suppressed the expression of *adcAI*. Growth in the presence of Cu alone suppressed *mtsC* expression (*P* < 0.0001). However, growth in the presence of Mn alone did not suppresss *mtsC* expression (*P* = 0.34).


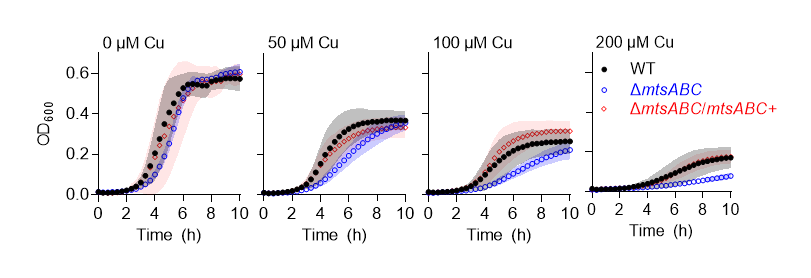


**Figure S5. Effects of Cu on bacterial growth of the ∆*mtsABC* mutant strain.** The GAS 5448 wild-type (filled circles), ∆*mtsABC* mutant (empty circles), and ∆*mtsABC*/*mtsABC^+^* complemented mutant (empty diamonds) strains were cultured with added Cu (0, 50, 100, or 200 µM) for *t* = 10 h (*N =* 3). Symbols represent means. Shaded regions represent SD. Cu treatment suppressed bacterial growth in general (*P* < 0.0001). The ∆*mtsABC* but not the ∆*mtsABC*/*mtsABC^+^* complemented mutant strain was more sensitive to inhibition by 200 µM Cu when compared with the WT (*P* < 0.0001 and *P =* 0.25, respectively).

**References**

(1) Chatellier, S.; Ihendyane, N.; Kansal, R. G.; Khambaty, F.; Basma, H.; Norrby-Teglund, A.; Low, D. E.; McGeer, A.; Kotb, M. Genetic Relatedness and Superantigen Expression in Group A Streptococcus Serotype M1 Isolates from Patients with Severe and Nonsevere Invasive Diseases. *Infect Immun* **2000**, *68* (6), 3523–3534.

(2) Stewart, L. J.; Ong, C. Y.; Zhang, M. M.; Brouwer, S.; McIntyre, L.; Davies, M. R.; Walker, M. J.; McEwan, A. G.; Waldron, K. J.; Djoko, K. Y. Role of Glutathione in Buffering Excess Intracellular Copper in Streptococcus Pyogenes. *mBio* *11* (6), e02804-20. https://doi.org/10.1128/mBio.02804-20.

(3) Ong, C. Y.; Berking, O.; Walker, M. J.; McEwan, A. G. New Insights into the Role of Zinc Acquisition and Zinc Tolerance in Group A Streptococcal Infection. *Infection and Immunity* **2018**, *86* (6). https://doi.org/10.1128/IAI.00048-18.

(4) Turner, A. G.; Djoko, K. Y.; Ong, C. Y.; Barnett, T. C.; Walker, M. J.; McEwan, A. G. Group A Streptococcus Co-Ordinates Manganese Import and Iron Efflux in Response to Hydrogen Peroxide Stress. *Biochemical Journal* **2019**, *476* (3), 595–611. https://doi.org/10.1042/BCJ20180902.
